# l-Carnitine Supplementation in Older Women. A Pilot Study on Aging Skeletal Muscle Mass and Function

**DOI:** 10.3390/nu10020255

**Published:** 2018-02-23

**Authors:** Angelika K. Sawicka, Dace Hartmane, Patrycja Lipinska, Ewa Wojtowicz, Wieslawa Lysiak-Szydlowska, Robert A. Olek

**Affiliations:** 1 Department of Bioenergetics and Nutrition, Gdansk University of Physical Education and Sport, 80-336 Gdansk, Poland; angelika.sawicka@awf.gda.pl; 2 Latvian Institute of Organic Synthesis, LV-1006 Riga, Latvia; dace_hartmane@osi.lv; 3 Institute of Physical Culture, Kazimierz Wielki University, 85-091 Bydgoszcz, Poland; patrycja.lipinska@ukw.edu.pl; 4 Department of Anatomy and Anthropology, Gdansk University of Physical Education and Sport, 80-336 Gdansk, Poland; ewawoj14@wp.pl; 5 Department of Health Sciences, Powislanski College, 82-500 Kwidzyn, Poland; wlysiak@onet.pl

**Keywords:** sarcopenia, cytokines, body composition, muscle strength

## Abstract

Skeletal muscle wasting, associated with aging, may be regulated by the inflammatory cytokines as well as by insulin-like growth factor 1 (IGF-1). l-carnitine possesses anti-inflammatory properties and increases plasma IGF-1 concentration, leading to the regulation of the genes responsible for protein catabolism and anabolism. The purpose of the present study was to evaluate the effect of a 24-week l-carnitine supplementation on serum inflammatory markers, IGF-1, body composition and skeletal muscle strength in healthy human subjects over 65 years of age. Women between 65 and 70 years of age were supplemented for 24 weeks with either 1500 mg l-carnitine-l-tartrate or an isonitrogenous placebo per day in a double-blind fashion. Before and after the supplementation protocol, body mass and composition, as well as knee extensor and flexor muscle strength were determined. In the blood samples, free carnitine, interleukin-6, tumor necrosis factor-α, C-reactive protein and IGF-1 were determined. A marked increase in free plasma carnitine concentration was observed due to l-carnitine supplementation. No substantial changes in other parameters were noted. In the current study, supplementation for 24 weeks affected neither the skeletal muscle strength nor circulating markers in healthy women over 65 years of age. Positive and negative aspects of l-carnitine supplementation need to be clarified.

## 1. Introduction

Aging is accompanied by a progressive change in the ratio between fat and lean body mass (BM). Fat mass, in particular visceral adipose tissue, increases, whereas fat free mass (FFM) declines [1]. Adipose tissue itself produces and releases a number of cytokines, such as interleukin-6 (IL-6), tumor necrosis factor-α (TNF-α), and other bioactive molecules [2]. In fact, even healthy aging results in slight elevations of circulating proinflammatory mediators, corresponding to a chronic low-grade inflammatory profile [3].

A body of literature has demonstrated that inflammatory cytokines activate many of the molecular pathways involved in skeletal muscle wasting, leading to an imbalance between protein synthesis and degradation [4,5]. High doses of TNF-α lead to reduced muscle cell differentiation in human and mouse muscle cells [6,7] and cause myotube (in vitro) and myofibre (in vivo) atrophy in animals [8,9], suggesting a predominant role for pro-inflammatory cytokine in reduced muscle regeneration, and thus a contributor to atrophy. Moreover, higher levels of IL-6 and TNF-α have been related with lower muscle strength and lower muscle mass in a cross-sectional study [10]. Cesari et al. [11], found associations of high levels of C-reactive protein (CRP) and IL-6 with poorer physical performance and muscle strength in elderly people. Longitudinal studies have shown elevated inflammatory markers as a predictor of increased incidence of mobility limitation [12] or the risk of muscle strength loss [13]. Skeletal muscle signaling pathways, which regulate anabolic and catabolic processes, may also be activated by insulin-like growth factor 1 (IGF-1) [4]. In cross-sectional studies of the European population recruited in five different countries, it has been demonstrated that circulating levels of IGF-1 decrease with age in both men and women [14].

l-carnitine is a low-molecular, nitrogenous compound, the main role of which is transporting long-chain fatty acids from the cytoplasm into the mitochondrial matrix [15]. It also possesses anti-inflammatory properties [16]. l-carnitine attenuates inflammatory changes in various experimental models: aging [17], liver fibrosis [18] or cancer cachexia [19]. Lee et al. [20] demonstrated that l-carnitine supplementation at a dose of 1000 mg/day for 12 weeks reduces the inflammatory status in the patients with coronary artery disease. The animal studies have indicated that l-carnitine suppresses the genes responsible for protein catabolism [21] and up-regulates the main drivers of protein anabolism [22] in skeletal muscle. Moreover, the activation of the signaling pathway is mediated by increased IGF-1 plasma concentration [22]. Furthermore, analysis of muscle samples of healthy humans of various ages shows a drastic reduction of l-carnitine and acetyl carnitine in the older subjects with a strong reverse correlation between age and l-carnitine levels [23]. Therefore, we hypothesized that l-carnitine supplementation for 24 weeks would affect the level of serum inflammatory markers, IGF-1, body composition and skeletal muscle strength in healthy human subjects over 65 years of age.

## 2. Materials and Methods

All subjects gave their informed consent for inclusion before they participated in the study. The study was conducted in accordance with the Declaration of Helsinki, and the protocol was approved by the Independent Bioethics Commission for Research at Medical University of Gdansk Ethics (NKBBN/354-304/2015).

### 2.1. Subjects

**Figure 1:**
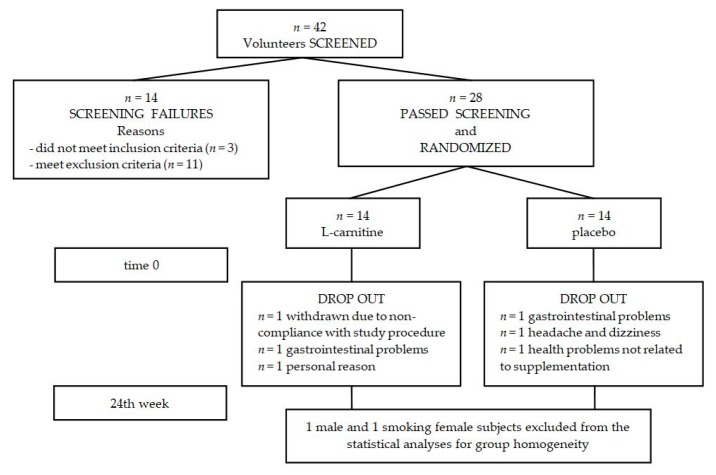
Disposition of study participants. A total of 42 participants were screened and 28 passed screening. The remaining 28 participants were enrolled in the study (14 in each group), but 22 completed the study while adhering to study protocols. One male subject and one smoking female subject were excluded from the statistical analyses for group homogeneity.

Forty-two subjects replied to the advertisements in the local newspaper at the University of Third Age and at the Senior Activity Center, and volunteered to participate in the study. Subjects with cancer, cardiovascular disease, gastrointestinal disease, liver, and renal diseases were excluded from the study. After the initial screening, 28 were included in the study and were randomly assigned to either an l-carnitine or a placebo supplementation group (Figure 1).

### 2.2. Study Procedure

Subjects were supplemented for 24 weeks with either 1500 mg l-carnitine-l-tartrate or a isonitrogenous placebo per day in a double-blind fashion. Supplements were kindly provided by Trec Nutrition Ltd. (Gdynia, Poland) and put inside identical gelatin capsules. Subjects were instructed to consume capsules daily after their main meal during the study period, because of the insulin-dependent l-carnitine transport into the skeletal muscle cell [24,25]. Before the start and after completion of the supplementation protocol, subjects arrived at the laboratory in the fasted state. Following weighting and blood sampling, standard light breakfasts were provided. Then, the set of laboratory tests were performed.

### 2.3. Anthropometric Measurements

Body mass and composition were estimated using a bioelectrical impedance analyzer, (InBody720, InBody Co., Ltd., Seoul, Korea). The participants had emptied their bladders and bowels prior to the measurement. The analyses were performed in the position recommended by the manufacturer’s guidelines and the subjects clad only underwear [26]. The InBody720 measures impedance of five segments of the body (each arm, each leg, trunk) at frequencies of 1, 5, 50, 250, 500, and 1000 kHz through the 8-polar tactile-electrode. Based on the impedance, FFM and skeletal muscle mass (SMM) were calculated.

### 2.4. Blood Sampling

Blood samples were drawn from a participant’s antecubital vein, maintaining a sterile field and using BD Vacutainer^®^ tubes (Becton, Dickinson and Company, Franklin Lakes, NJ, USA) with serum-separator or ethylenediaminetetraacetic acid (EDTA). After collection, the samples were centrifuged at 2000× *g* at 4 °C for 10 min. Aliquots were stored at −80 °C for later analyses.

### 2.5. Biochemical Determination

Plasma free carnitine was determined by the ultra-high-performance liquid chromatography-mass spectrometry (UPLC/MS/MS) method as previously described [27]. Serum IL-6, TNF-α, CRP and IGF-1 concentrations were determined by the enzyme immunoassay method using commercially available kits (IL-6, cat. no. HS600B; TNF-α, cat. no. HSTA00D; total IGF-1, cat. no. DG100—R&D Systems, Minneapolis, MN, USA; CRP, cat. no. SEA821Hu—Cloud-Clone Corp., Houston, TX, USA).

### 2.6. Skeletal Muscle Strength Test

Knee extensor and flexor muscle strength were determined by a Biodex System 4 Pro™ dynamometer (Biodex Medical Systems, Inc., Shirley, NY, USA). After a warm-up, subjects were positioned according to the manufacturer’s manual (seated with arms hanging along the body, hands holding the lateral handles, and strap stabilization of trunk, hip, and tested thigh, with the knee flexed at 90°) [28]. The testing range of motion was 80° and was set from 10–90° of knee flexion (with 0° = full voluntary extension). The seat position was adjusted for the leg length of each tested person. The dominant leg was examined using isokinetics tests. The isokinetic knee flexion and extension was measured at angular velocities of 60°/s [29]. All the measurements were normalized by the lean mass of the working leg.

### 2.7. Nutritional and Physical Activity Habits

Nutritional intake patterns, especially red meat, have a great impact on total body carnitine content [30]. Therefore, to assess the frequency of meat consumption, a specific survey was designed in a way that was both simple to complete and easily comprehensible. The survey used quantitative research methods to identify six “frequency consumption groups” [31]:

F0—never,
F1—occasionally,
F2—several times per year,
F3—several times per month,
F4—2–5 times per week,
F5—6–7 times per week.

The short form of the International Physical Activity Questionnaire (IPAQ) was used for the assessment of habitual physical activity. The following metabolic equivalent (MET) values were used for the analysis of IPAQ: walking = 3.3 METs, moderate physical activity = 4.0 METs, vigorous physical activity = 8.0 METs [32]. The total physical activity of the subjects was classified as low, moderate or high. Subject characteristics are presented in Table 1.

### 2.8. Statistical Analyses

Basic anthropometric characteristics of the subjects were evaluated as mean ± SD. For educational and physical activity levels, a percentage of the total tested was used, while a frequency of meat consumption was presented as median and range. Changes in both groups across the supplementation time and ratios as well as changes in other measurements were analyzed with a Microsoft Excel (Microsoft Office Home and Student 2007, Version 12.0.6612.1000, Microsoft, Redmond, WA, USA) spreadsheet for the analysis of parallel-group controlled trials [33]. For this, effects were interpreted using magnitude-based inferences [34]. All data were log-transformed for analysis to reduce bias arising from non-uniformity of error; means of change scores in the placebo and l-carnitine groups, standard deviations of change scores, and effects (variations of change in both the means and their confidence limits (CL)) were back-transformed to percent units. Mean changes and effects were adjusted to the overall mean baseline value of the placebo and l-carnitine groups, by including the baseline value as a covariate in the analysis. Magnitudes of the effects were evaluated with the log-transformed data by standardizing the deviation of the baseline values of the placebo and l-carnitine groups. Threshold values for assessing magnitudes of standardized effects were 0.20, 0.60, 1.2 and 2.0 for small, moderate, large and very large respectively. Uncertainty in the effects was expressed as 90% CL and as probabilities that the true value of the effect was beneficial, trivial or harmful. These probabilities are not presented quantitatively but were used to make qualitative probabilistic clinical inferences about effects in preference to a statistical inference based on a null-hypothesis significance test [34]. The effect was deemed unclear when the chance of benefit was sufficiently high to warrant the use of the treatment but the risk of harm was unacceptable. Such unclear effects were identified as those with an odds ratio of benefit to harm of <66, a ratio that corresponds to an effect that is borderline possibly beneficial (25% chance of benefit) and borderline most unlikely harmful (0.5% risk of harm). All other effects were deemed clinically clear and the likelihood of the true effect as being trivial, beneficial or harmful was expressed with the following scale: 25–75%, possibly; 75–95%, likely; 95–99.5%, very likely; >99.5%, most likely. To maintain an overall error rate of <5% to declare one or more changes as having opposite magnitudes (a substantial decrease instead of an increase, and vice versa), the effects were also evaluated as beneficial or harmful with a threshold of 1%, equivalent to the consideration of the overlap of substantial values with a 98% confidence interval (CI).

## 3. Results

In the study, participants were all non-smoking, non-obese, non-vegetarian, physically active women in the age range of 65 to 70 years. The ratio of percent changes in BM at baseline and across a supplementation period in placebo and l-carnitine groups was most likely trivial (0.5%; ±1.9%; mean; 90% CL). The effects were clear, which indicates that supplementation did not affect BM. Ratios of percent changes in BM components (FFM and SMM) were likely trivial (clear effects, 0.7%; ±2.6%; 1.2%; ±3.2%, respectively) (Table 2).

Baseline measures of circulating parameters, their percent changes across a supplementation period in both groups, and ratios of the changes are presented in Table 3.

A marked increase in free plasma carnitine concentration was observed due to l-carnitine supplementation. Other ratios of changes, with the exception of unclear change in CRP, were possibly trivial (Table 3).

No substantial changes in the isokinetic measures were noted in response to the supplementation period (Table 4).

## 4. Discussion

Systemic l-carnitine depletion has been described in aging, and is characterized by fatigue, muscle wasting, and geriatric frailty [35]. In the current study, we have evaluated the effect of l-carnitine supplementation on muscle mass, strength, and selected blood markers in older women at risk for sarcopenia.

In a recent meta-analysis, Pooyandjoo et al. [36] calculated that subjects supplemented with l-carnitine lost significantly more weight (−1.33 kg), compared with the control group. This article was later criticized [37] for mixing studies using l-carnitine alone or in association with other factors (i.e., pharmacological therapy-sibutramine [38] or orlistat [39] and changes in lifestyle–diet intervention [40]). Inclusion of the studies using treatments consisting exclusively of l-carnitine indicated no modification in body weight [37]. Twenty-four weeks of l-carnitine supplementation, without changing nutritional or physical activity habits, did not influence our participants’ body weight. Interestingly, participants supplemented with l-carnitine maintained a similar SMM from baseline to 24 weeks, while the placebo group showed a decline in SMM within the population range—an approximate annual rate of 1 to 2% [41]. However, due to the variability within the groups themselves, the effect between the two groups was trivial. In a previous study, Malaguarnera et al. [42] supplemented the diet of centenarians with 2 g of l-carnitine per day. Six months of intervention induced a significant increase in FFM in comparison with pre-treatment values, and in comparison with post-treatment values in the placebo group [42]. However, the mechanism has not been proposed.

Muscle wasting in sarcopenia populations is associated with a shift from muscle protein synthesis to muscle protein degradation. The mechanism of muscle wasting may involve low-grade inflammation [4,5]. It has been proposed that circulating cytokines and CRP negatively affect muscle mass during aging [10,11]. Meta-analysis of human studies suggests potential beneficial effects of l-carnitine in lowering the circulating CRP level [43]. However, the magnitude of significant effect of l-carnitine intervention is based on the results of the trials with an initial CRP level > 5 mg/L [40,44,45]. Be that as it may, l-carnitine treatment does not reveal the anti-inflammatory properties in low-grade inflammation conditions [46,47]. No alterations in serum CRP, IL-6 and IL-10 have been reported in obese women following an eight-week supplementation of 2 g l-carnitine per day [47]. Similarly, in our study, the elevation of plasma-free l-carnitine concentration after a 24-week supplementation period did not affect the circulating proteins and was not accompanied by the anti-inflammatory effect.

The muscle protein synthesis and degradation rate is multifactorial and includes various signaling pathways [48,49]. The animal studies show that supplementation of l-carnitine elevates plasma IGF-1 concentration, despite various doses consumed daily: from ~1 mg/kg body weight in sows [50], to ~100 mg/kg body weight in rats [22]. The binding of IGF-1 to its receptor triggers the activation of the protein synthesis pathway phosphatidylinositol 3-kinase (PI3K)/protein kinase B (Akt)/mammalian target of rapamycin (mTOR) [51], which is up-regulated during hypertrophy and down-regulated during muscle atrophy [52]. The IGF-1/PI3K/Akt signaling pathway is not only capable of activating anabolic pathways, but also of suppressing catabolic pathways, due to an inactivation of a specific transcription factor family called forkhead box-O transcription factors (FOXO) [53]. l-carnitine supplementation may affect both of these pathways in rat skeletal muscle [22]. Human studies show a decrease in circulating IGF-1 with aging [14], which is in agreement with our observation. However, l-carnitine caused a trivial attenuation in plasma IGF-1 level decline compared to the placebo.

Recent human aging studies present the increase in skeletal muscle mass and strength due to the mTOR pathway activation, following an eight-week supplementation of l-carnitine when combined with creatine, l-leucine and vitamin D [54]. However, these effects are not present in the group supplemented only by the same dose of l-carnitine without any additional supplements [54]. Therefore, it cannot be ruled out that other supplements affect sarcopenia by their own properties; creatine delays muscle atrophy and improves strength in aging [55], l-leucine increases the muscle protein fractional synthesis rate in the elderly individuals [56], and vitamin D improves muscle strength in people ≥ 65 years of age [57]. A similar dose of l-carnitine was used in our study, without any additional supplements, but for a prolonged period of time and did not significantly affect muscle mass and function.

The quadriceps muscle strength declines in aging women in correlation to the muscle cross sectional area [58]. Therefore, in order to obtain better relative values, we normalized total performed work, peak torque and power per lean mass of the working leg. The longitudinal study shows losses of 11.8% per decade in women’s knee extensor strength and 17.4% per decade in flexor strength [59]. It is noteworthy that in men, these values showed no reduction in muscle strength parameters. The lack of changes in muscle strength may have been influenced by physical activity, since the group of participants was characterized by relatively high physical activity.

## 5. Conclusions

l-carnitine is a popular nutritional supplement, which may be beneficial in some pathological conditions characterized by chronic systemic inflammation and muscle wasting [60]. In the current study, supplementation for 24 weeks did not affect either the skeletal muscle strength or circulating markers in healthy women over 65 years of age. Positive and negative aspects of l-carnitine supplementation need to be clarified, especially considering its metabolite trimethylamino-*N*-oxide, which may be associated with the etiology of some diseases [61].

## Figures and Tables

**Table 1:** Basic characteristics of the participants.

Variables	Placebo		l-Carnitine	
	Mean ± SD (Standard Deviation)		Mean ± SD	
Age (years)	66.4 ± 1.3		67.8 ± 2.3	
Height (cm)	162 ± 5.3		159 ± 5.4	
BMI (kg/m^2^)	26.5 ± 4.4		27.5 ± 4.5	
	*n*	%	*n*	%
Education level				
Primary	0	0	0	0
Secondary	2	22.2	4	36.4
High	7	77.8	7	63.6
Physical activity				
Low	0	0	0	0
Moderate	4	44.4	6	54.5
High	5	55.6	5	45.5
	median	range	median	range
Meat consumption				
Poultry	F4	F3–F4	F4	F0–F4
Pork	F2	F0–F3	F3	F0–F4
Beef	F1	F0–F3	F2	F0–F4
Fish	F3	F1–F4	F2	F1–F4
Lamb	F0	F0–F3	F0	F0–F3
Venison	F0	F0–F3	F0	F0–F1
Horseflesh	F0	F0–F2	F0	F0–F1

**Table 2:** Baseline and changes in the measures across a supplementation period in both groups.

Variables	Group	Baseline Mean ± SD	Observed Change Mean ± SD	Adjusted Change ^a^ Mean ± SD	Adjusted Effect ^b^	
					Mean; CL	Inference
BM (kg)	placebo	69.7 ± 12.1	−0.3 ± 2.8%	−0.3 ± 2.1%	0.5%; ±1.9%	trivial ^†^
	l-carnitine	69.8 ± 12.9	0.2 ± 2.9%	0.2 ± 2.9%		
FFM (kg)	placebo	45.8 ± 6.7	−1.6 ± 5.2%	−1.1 ± 3.7%	0.7%; ±2.6%	trivial *
	l-carnitine	43.9 ± 4.6	0.0 ± 3.5%	−0.4 ± 2.8%		
SMM (kg)	placebo	24.9 ± 4.1	−1.9 ± 6.4%	−1.3 ± 4.4%	1.2%; ±3.2%	trivial *
	l-carnitine	23.9 ± 2.7	−0.2 ± 3.8%	−0.1 ± 3.2%		

CL, 90% confidence limit; ^†^ most likely, * possible. ^a^ Adjusted to the overall mean of both groups at baseline. ^b^ Adjusted mean change in the l-carnitine group minus the adjusted mean change in the placebo group. BM: body mass; FFM: fat free mass; SMM: skeletal muscle mass.

**Table 3:** Baseline and changes across the supplementation period in both groups and ratios of changes as effects.

Variables	Group	Baseline Mean ± SD	Observed Change Mean ± SD	Adjusted Change ^a^ Mean ± SD	Adjusted Effect ^b^	
					Mean; CL	Inference
free carnitine (µmol/L)	placebo	39.5 ± 3.7	10 ± 11%	8 ± 6%	13%; ±5.8%	moderate ^†^
	l-carnitine	41.1 ± 6.4	22 ± 9%	22 ± 8%		
CRP (mg/L)	placebo	1.8 ± 0.8	−6 ± 15%	−4.7 ± 15%	21%; ±37%	unclear
	l-carnitine	2.6 ± 1.1	8 ± 68%	16 ± 65%		
IL-6 (ng/L)	placebo	1.8 ± 0.7	−10 ± 23%	−13 ± 20%	4.9%; ±22%	trivial *
	l-carnitine	2.2 ± 1.1	−11 ± 42%	−8.2 ± 32%		
TNF (ng/L)	placebo	0.56 ± 0.26	14 ± 70%	12 ± 68%	9.0%; ±50%	trivial *
	l-carnitine	0.58 ± 0.32	24 ± 82%	28 ± 38%		
IGF-1 (µg/L)	placebo	78 ± 19	−10 ± 12%	−10 ± 13%	1.8%; ±16%	trivial *
	l-carnitine	69 ± 15	−6 ± 28%	−8 ± 28%		

CL, 90% confidence limit; ^†^ most likely; * possible, underlined effect is also clear at the 0.5% level (98% confidence interval). ^a^ Adjusted to the overall mean of both groups at baseline. ^b^ Adjusted mean change in the l-carnitine group minus the adjusted mean change in the placebo group. CRP: C-reactive protein; IL-6: interleukin 6; TNF-α: tumor necrosis factor-alpha; IGF-1: insulin-like growth factor 1.

**Table 4:** Isokinetic measures for the dominant leg at baseline, and changes across a supplementation period in both groups.

Variables	Group	Baseline Mean ± SD	Observed Change Mean ± SD	Adjusted Change ^a^ Mean ± SD	Adjusted Effect ^b^	
					Mean; CL	Inference
TW extension (J/kg)	placebo	76 ± 15	7.4 ± 26%	5.7 ± 8.3%	5.6%; ±7.1%	trivial *
	l-carnitine	78 ± 11	11 ± 13%	12 ± 9.5%		
TW flexion (J/kg)	placebo	43 ± 17	9.4 ± 40%	13 ± 13%	−2.9%; ±13%	trivial *
	l-carnitine	36 ± 9	14 ± 34%	9.7 ± 23%		
APT extension (Nm/kg)	placebo	12.0 ± 2.6	4.7 ± 20%	1.9 ± 9.7%	3.0%; ±9.0%	unclear
	l-carnitine	12.8 ± 2.3	2.7 ± 18%	5.0 ± 13%		
APT flexion (Nm/kg)	placebo	6.8 ± 2.6	4.9 ± 26%	7.0 ± 7.8%	−4.2%; ±9.0%	trivial *
	l-carnitine	5.9 ± 1.1	5.0 ± 22%	2.6 ± 16%		
AP extension (W/kg)	placebo	8.3 ± 2.0	6.3 ± 25%	3.9 ± 6.2%	1.4%; ±6.8%	trivial ^†^
	l-carnitine	8.6 ± 1.4	3.3 ± 18%	5.4 ± 11%		
AP flexion (W/kg)	placebo	4.4 ± 1.7	10 ± 40%	14 ± 12%	−7.1%; ±9.7%	trivial ^†^
	l-carnitine	3.9 ± 1.4	12 ± 83%	5.8 ± 17%		

CL, 90% confidence limit; * possible, ^†^ likely. ^a^ Adjusted to the overall mean of both groups at baseline. ^b^ Adjusted mean change in the l-carnitine group minus the adjusted mean change in the placebo group. TW: total work; APT: average peak torque; AP: average power.
